# Current status and prospect of particle therapy for esophageal cancer

**DOI:** 10.1002/pro6.1232

**Published:** 2024-06-27

**Authors:** Kang Wang, Shuanghu Yuan

**Affiliations:** ^1^ Department of Radiation Oncology Shandong Cancer Hospital and Institute Shandong First Medical University and Shandong Academy of Medical Sciences Jinan Shandong China; ^2^ Department of Radiation Oncology First Affiliated Hospital of USTC Division of Life Sciences and Medicine University of Science and Technology of China Hefei Anhui China

**Keywords:** Esophageal cancer, proton therapy, carbon‐ion therapy, radiotherapy

## Abstract

Esophageal cancer is among the top causes of cancer‐related mortality worldwide, and the main treatment modality for locally advanced esophageal cancer is concurrent chemoradiotherapy. The current photon‐based radiotherapy modalities and procedures have increased the incidence of treatment‐related cardiac and pulmonary complications. Additionally, anatomical changes in the esophagus resulting from diaphragmatic movement, weight loss, and tumor progression present challenges for radiotherapy. These challenges have spurred interest in particle therapies, such as proton beam therapy (PBT) and heavy‐ion therapy, for esophageal cancer. This paper comprehensively reviews the dosimetric advantages, clinical efficacy, and limitations of PBT and heavy‐ion therapy for esophageal cancer and discusses their prospects. This highlights the unique dosimetric benefits of these therapies, particularly their ability to deliver high‐dose radiation precisely to the tumor while sparing the surrounding normal organs and tissues. Although PBT and heavy‐ion therapy demonstrate superior clinical efficacy compared to photon therapy, they are not without limitations. Multiple studies are needed to further validate and supplement the existing clinical and preclinical data to better exploit the benefits of PBT and thereby provide improved survival advantages to these patients

## Graphical abstract

Dosimetric advantages of PBT as treatment for esophageal cancer

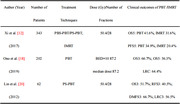

## 1 INTRODUCTION

Esophageal cancer is one of the most common digestive system tumors. In 2020, esophageal cancer was ranked seventh in global incidence, with 604,100 new cases (3.1%) reported. Additionally, it ranked sixth in mortality among all cancers, with even developed countries exhibiting a 5‐year survival rate of ≤ 40%.^1^, ^2^ The incidence of esophageal cancer exhibits geographic variability, particularly that of esophageal squamous cell carcinoma, which has the highest prevalence in Asia.^3^ Neoadjuvant radiotherapy followed by surgery is the standard treatment option for patients with operable locally advanced esophageal cancer, as supported by a meta‐analysis and the CROSS study.^4^ Concurrent chemoradiotherapy (cCRT) is a curative option for patients with unresectable locally advanced esophageal cancer, and postoperative adjuvant radiotherapy can improve local control and survival in selected cases.^5^, ^6^

With the development of computed tomography (CT), advances in X‐ray‐based conventional radiotherapy have greatly improved its therapeutic effect. Two‐dimensional radiotherapy has progressively transitioned to three‐dimensional conformal radiotherapy (3D‐CRT) and has further evolved into modern techniques such as volumetric‐modulated arc therapy (VMAT), image‐guided radiotherapy, and intensity‐modulated radiotherapy (IMRT).^7^ IMRT can preserve adjacent normal tissues to a certain extent without affecting the dose to the tumor target. However, current photon‐based radiation therapy is still prone to complications owing to the involvement of adjacent organs in esophageal malignancies.^8^ Anatomical changes, such as variations in position, diaphragmatic movement, weight loss, and tumor dynamics, present significant challenges in the delivery of radiation therapy for esophageal cancer. These challenges have spurred advancements in proton beam therapy (PBT) and heavy ion therapy for esophageal cancer. This review examines the present state and prospects of PBT and heavy‐ion therapy in the treatment of esophageal cancer with the goal of offering new directions and ideas to enhance the effectiveness of radiation therapy for this disease.

## 2 DOSIMETRIC ADVANTAGES OF PBT AS A TREATMENT FOR ESOPHAGEAL CANCER

Despite attenuation, conventional X‐rays result in physically insurmountable radiation exposure doses, leading to significant radiation doses in normal tissues beyond the tumor. The esophagus is anatomically situated in the central region of the mediastinum and is enveloped by both the lungs and mediastinal structures.^9^ PBT was initially proposed in 1946 by Robert R. Wilson, who believed that the distinctive physical properties of protons could offer advantages for treating deep‐seated tumors.^10^ Protons, which are heavy, charged particles, can be derived from hydrogen, and accelerated to the speed of light using a cyclotron or synchrotron. The proton beams are used to treat tumors in a manner similar to conventional X‐ray radiation therapy. Theoretically, PBTs deposit their energy at the end of their path, peaking at the tumor site owing to its characteristic Bragg peak model. This allows it to deliver sufficient radiation dose to the target tumor followed by rapid dose reduction, resulting in a minimal exit dose.^7^ The depth of the Bragg peak is energy‐dependent and can be adjusted by modulating the energy and expanding the width of the peak according to the volume of the tumor. This enables the high‐energy area to be concentrated at different depths and sizes of the tumor site, significantly increasing the therapeutic dose to the tumor.^11^ Because of the low flatness of the pre‐peak energy release, the normal tissues superficial to the tumor located in the direction of the proton beam are exposed to only 1/3 to 1/2 of the peak dose, whereas the normal tissues deeper to the tumor are essentially undamaged. This unique dose‐attenuation property reduces adverse radiation effects in patients, which is particularly important when the tumor is adjacent to critical organs.^12^ Protons in the human body propagate linearly and have a relatively large mass with minimal scattering, thus reducing the irradiation dose to the surrounding normal tissues and improving the accuracy of tumor treatment. Automation technology can control the direction, location, and range of the proton beam energy release, positioning the Bragg peak at the tumor target to achieve precise targeting. Simultaneously, it reduces the radiation exposure to neighboring healthy tissues, thereby enhancing the quality of patient survival.

Studies have demonstrated that PBT offers distinct dosimetric advantages over photon therapy for the treatment of esophageal cancer, leading to a significant reduction in radiation‐induced toxicity to the surrounding normal organs and tissues. Parzen et al.^13^ analyzed 155 patients diagnosed with esophageal cancer who underwent PBT at 10 institutions between 2010 and 2019. Among them, 120 (77%) had adenocarcinomas and 34 (22%) had squamous cell carcinomas, and received a median radiation dose of 50.51 Gy (range 41.4‐70.1 Gy). Twenty‐two patients (14%) experienced grade 3 or higher toxic reactions, primarily presenting as dysphagia (6%), esophagitis (4%), loss of appetite (4%), and nausea (2%). No cases of grade ≥ 4 lymphopenia or grade 5 toxic reactions were observed. The mean doses to the heart and liver were 10.5 Gy and 3.9 Gy, respectively. Xi et al.^14^ conducted a retrospective study involving 343 patients and compared IMRT and PBT treatments using the posterior and left posterior oblique fields. The study revealed that the mean doses to the lungs and heart in the IMRT technique were 10.0 Gy and 19.9 Gy, respectively, which were significantly higher than those of the PBT group (6.5 Gy and 11.6 Gy, respectively). Furthermore, the PBT group exhibited significantly lower lung V_5_ (28% vs. 48%) and V_10_ (23% vs. 32%), as well as lower cardiac V_30_ (19% vs. 24%) than that of the IMRT group.

Specialized proton beam facilities are required to safeguard critical organs during the treatment of esophageal cancer. Oonsiri et al.^15^ proposed a novel beam arrangement for intensity‐modulated proton therapy (IMPT) and compared it with intensity‐modulated X‐ray therapy (IMXT). They demonstrated that while IMPT provided comparable target coverage to IMXT, it significantly reduced the mean dose to the organs at risk. Specifically, IMPT using the anterior‐posterior opposing beam arrangement yielded the lowest lung dose (mean lung dose = 7.1 Gy, V_20_ = 14.1%), and IMPT using the anterior‐superior, and posterior‐inferior beam resulted in the lowest cardiac dose (mean heart dose = 12.8 Gy, V_30_ = 15.7%). Notably, in patients with tumors in the inferior location, IMPT exhibited superior protection of critical organs. Hirano et al.^16^ investigated 27 patients with clinical stage III esophageal cancer and conducted a dosimetric analysis comparing pencil beam scanning PBT (PBS‐PBT), 3D‐CRT, and IMRT. They observed that PBS‐PBT reduced the dose to organs at risk, particularly the lungs and heart. Of these patients, four (15%) developed grade 2 pericardial effusions, and no grade 3 or worse acute or late pulmonary and cardiac toxicity was reported. Furthermore, when comparing the dosimetric parameters of PBS‐PBT, 3D‐CRT, and IMRT, the pulmonary and cardiac doses were significantly lower with PBS‐PBT than with 3D‐CRT and IMRT for all parameters, except for lung V_10_ and V_15_.

Esophageal radiotherapy can result in various toxicities. Myelosuppression is the most common acute toxicity observed and is caused, in part, by the action of radiation on the vertebrae immediately posterior to the esophagus, especially in the lower thoracic esophagus. Zhang et al.^17^ compared passive or double‐scattering PBT (PS‐PBT) with VMAT and IMRT for treating patients with locally advanced esophageal cancer. PS‐PBT was found to significantly reduce the vertebral dose compared to VMAT and IMRT. This reduction in the vertebral dose can consequently mitigate treatment‐related myelosuppression, prevent treatment delays or dose reductions in chemotherapy, and enhance both the quality of life and survival rate of patients. Table 1 presents a comparison of the dosimetric characteristics and toxicities of PBT and photon beam therapy.

**TABLE 1:** Dosimetric studies comparing photon‐based and proton beam therapy techniques for esophageal cancer treatment.

Author (Year)	Number of Patients	Treatment Techniques	Dose (Gy)/Number of Fractions	Lung Toxicity	Heart Toxicity	Other Toxicities
Parzen et al.^13^ (2021)	155	PBS‐PBT/PS‐PBT	‐	Average mean total lung dose: 4.8 GyE (range, 1.4‐13.4)	Average mean heart dose: 10.0 GyE (range, 1.4‐25.9)	Radiation dermatitis 65%; fatigue 60%; nausea 56%; anorexia 43%; esophagitis 40%; dysphagia 29%
Xi et al.^14^ (2017)	343	PBS‐PBT/PS‐PBT vs. IMRT	50.4/28.0	Grade ≥3: Pneumonitis 2.9% IMRT, 1.6% PBT; Pleural effusion 1.9% IMRT, 0.8% PBT	Grade ≥3: Pericardial effusion 2.4% IMRT, 0.8% PBT	Grade ≥3: Esophagitis 14.2% IMRT, 11.4% PBT; Nausea 7.1% IMRT, 6.8% PBT; Esophageal stricture 8.1% IMRT, 9.8% PBT
Oonsiri et al.^15^ (2022)	25	IMXT vs. IMPT	Low‐risk target volumes: 50–54 Gy/high‐risk target volumes: 60–64 Gy	IMPT with an anteroposterior opposing beam generated the lowest lung dose (mean = 7.1 Gy (RBE))	IMPT: the anterosuperior with posteroinferior beam resulted in the lowest heart dose (mean = 12.8 Gy (RBE))	IMPT: the anterosuperior with posteroinferior beam resulted in the lowest liver dose (mean = 3.9 Gy (RBE))
Hirano et al.^16^ (2018)	27	PBS‐PBT vs. 3D‐CRT or IMRT	60/30	Grade ≥1: PBT Pneumonitis: Acute toxicity 26%; Late toxicity 63%	Grade ≥2: PBT Pericardial effusion: Acute toxicity 4%; Late toxicity 15%	‐
Bhangoo et al.^18^ (2020)	64	IMPT vs. IMRT	45/25	Pneumonia: 28% IMRT, 13% IMPT; ARDS: 17% IMRT, 0% IMPT	Cardiac arrhythmia: 17% IMRT, 53% IMPT	Esophageal stricture: 11% IMRT, 7% IMPT; Anastomotic leak: 22% IMRT, 20% IMPT; Anastomotic stricture: 39% IMRT, 27% IMPT
Lin et al.^19^ (2017)	580	PBT vs. IMRT vs. 3D‐CRT	‐	Pulmonary complications: 39.5% 3D‐CRT, 24.3% IMRT, 16.2% PBT	Cardiac complications: 27.4% 3D‐CRT, 11.7% IMRT, 11.7% PBT	Wound complications: 15.3% 3D‐CRT, 14.1% IMRT, 4.5% PBT

Abbreviations: IMXT, intensity‐modulated X‐ray therapy; IMPT, intensity‐modulated proton therapy; RBE, relative biological effectiveness; PBS‐PBT, pencil beam scanning proton beam therapy; PS‐PBT, passive scattering proton beam therapy; IMRT, intensity‐modulated radiotherapy; 3D‐CRT, three‐dimensional conformal radiotherapy; PBT, proton beam therapy.

Overall, PBT outperformed photon therapy in minimizing doses to the lungs, heart, and vertebrae. However, it is important to note that the unique dose distribution characteristics of PBT necessitate stringent program stability and accuracy. Failure to maintain these standards may result in significant adverse effects on organ‐at‐risk protection and target volume dose coverage.

## 3 CLINICAL EFFICACY CHARACTERISTICS OF PBT IN THE TREATMENT OF ESOPHAGEAL CANCER

Dosimetric data support the superiority of PBT for the treatment of esophageal cancer. However, data on the prognostic status of patients with esophageal cancer undergoing PBT are limited. Specifically, there is a scarcity of prospective studies directly comparing PBT with photon radiotherapy. Ono et al.^20^ analyzed the clinical outcomes of 202 patients with esophageal cancer who underwent PBT. The cohort comprised 72 patients (35.6%) in stage I, 30 (14.9%) in stage II, 52 (25.7%) in stage III, and 48 (23.8%) in stage IV. The median total dose was 87.2 Gy. The overall survival (OS) rates at 3 and 5 years were 66.7% and 56.3%, respectively. The 5‐year OS rates for stage I, II, III, and IV were 79.3%, 66.3%, 43.2%, and 28.3%, respectively. No grade 4 or higher toxicity events were recorded. Another study by the same authors^21^ analyzed patients aged 75 years and older with squamous cell carcinoma of the esophagus who received PBT. More than 40% of the patients had stage III/IV disease. The 5‐year locoregional control (LRC) rate was 61.8% and the local recurrence rate was 82.4% within 13 months. No grade 3 or higher toxicities were observed, except for grade 3 esophageal ulcers in 3 patients. Lin et al.^22^ conducted a phase IIB clinical trial on locally advanced esophageal cancer, randomly assigning 145 patients to either the IMRT (72 patients) or PBT (73 patients) groups. The median follow‐up was 44.1 months. The 3‐year progression‐free survival (PFS) rate was 44.5% for both IMRT and PBT. However, the 3‐year OS rates for IMRT and PBT were 50.8% and 51.2%, respectively. PBT yielded significantly lower doses to the total lung parameters (V_5_, 41.4% vs. 19.7%; V_20_, 13.6% vs. 8.4%; mean lung dose, 8.4 vs 4.8 Gy; P < 0.001 for all), as well as mean doses to the heart (19.8 vs 11.3 Gy; P < 0.001) and liver (12.1 vs 2.4 Gy; P < 0.001), but similar maximum spinal cord doses (38.4 vs 38.3 Gy; P = 0.47). PBT for neoadjuvant or radical treatment of locally advanced esophageal cancer was less toxic than IMRT; however, both treatments had similar PFS. Despite the limited clinical data on PBT for esophageal cancer, the above studies indicate its efficacy in enhancing the prognosis of patients with esophageal cancer compared with conventional photon radiotherapy.

Xi et al.^14^ conducted a retrospective comparison of survival outcomes in patients with esophageal cancer treated with PBT (n = 132) versus IMRT (n = 211). The median dose was 50.4 Gy for both treatment modalities. Among the PBT group, 94.7% were treated with PS‐PBT, and only seven patients (5.3%) received IMPT. The results showed that PBT resulted in significantly better OS than IMRT, particularly in patients with stage III esophageal cancer. The 5‐year OS rates were 34.6% for PBT and 25.0% for IMRT, and the 5‐year PFS rates were 33.5% and 13.2%, respectively. Lin et al.^23^ presented findings from another study involving 62 patients with esophageal cancer who underwent PBT. The prescription dose was 50.4 Gy in 28 fractions. The cohort comprised 47 (75.8%) patients with adenocarcinoma and 14 (22.6%) with squamous cell carcinoma, with the majority of patients (84%) classified as having stage II‐III disease. Radical radiotherapy was administered to 33 patients (53.2%), and 29 patients (46.8%) underwent surgery following induction therapy. The pathological complete remission rate after surgical resection following radiochemotherapy was 28%. PBT was well tolerated, with a grade 2–3 pneumonitis incidence rate of 3.2%. Radical radiotherapy achieved 3‐year OS and LRC rates of 51.7% and 56.5%, respectively. These studies demonstrate that the dose advantage of PBT over IMRT translates into improved clinical outcomes. PBT for esophageal cancer may prolong survival by delivering higher doses without additional toxicity. Table 2 presents a comparison of the clinical outcomes of PBT and photon beam therapy.

**TABLE 2:** Clinical studies comparing PBT and IMRT for esophageal cancer treatment.

Author (Year)	Number of Patients	Treatment Techniques	Dose (Gy)/Number of Fractions	Clinical outcomes of PBT /IMRT
Xi et al.^14^ (2017)	343	PBS‐PBT/PS‐PBT; IMRT	50.4/28	OS‐5: PBT 41.6%, IMRT 31.6%; PFS‐5: PBT 34.9%, IMRT 20.4%
Ono et al.^20^ (2019)	202	PBT	BED = 10 87.2 median dose: 87.2	OS‐3: 66.7%; OS‐5: 56.3% LRC: 64.4%
Lin et al.^23^ (2012)	62	PS‐PBT	50.4/28	OS‐3: 51.7%; RFS‐3: 40.5%; DMFS‐3: 66.7%; LRC‐3: 56.5%

*Note*: This table builds upon the studies reviewed by Jethwa et al.^24^ (2020)

Abbreviations: BED, biologically effective dose; DMFS‐3, 3‐year distant metastasis‐free survival; IMRT, intensity‐modulated radiotherapy; LRC‐3, 3‐year LRC, locoregional control; LRC, locoregional control; OS‐3, 3‐year overall survival; OS‐5, 5‐year overall survival; PBS‐PBT, pencil beam scanning proton beam therapy; PBT, proton beam therapy; PFS‐5, 5‐year progression‐free survival; PS‐PBT, passive‐scattering proton beam therapy; RFS‐3, 3‐year recurrence‐free survival.

## 4 LIMITATIONS OF PBT IN THE TREATMENT OF ESOPHAGEAL CANCER

The proton beam dose distribution is significantly influenced by variations in the tissue material and density. The anatomy of the patient can change due to diaphragmatic motion, cyclic tumor movement, weight loss, and pleural effusion. Proton beams deliver dose to the esophagus in spatially discrete “spots” within minutes, resulting in dose inhomogeneity within the target volume particularly influenced by respiratory motion.^25^ Moreover, esophageal cancer target regions are primarily situated in the chest and upper abdomen and exhibit complex tissue distribution. Variations in tissue density along the path of the proton beams can lead to inadequate or excessive dosing of the target volume in normal tissues.^26^ Owing to these limitations, PBT may not be appropriate for all patients, particularly those with tumors located in the gastroesophageal junction. While technological advancements, such as four‐dimensional computed tomography‐based motion management, robustness optimization and assessment, active motion management, rescanning, tracking, and adaptive planning, can mitigate the associated uncertainties, the dose distribution remains inherently unstable.^27^ Various methods exist for tracking respiratory motion. One promising approach is the use of an infrared camera to monitor the temperature changes of the air entering and leaving the nostrils of the patient. This method enables the quantification of the tidal volume, which can be accurately correlated with respiratory motion and subsequently used to synchronize proton beam delivery.^28^

High cost is also a significant limitation of PBT, as it requires extensive space and incurs higher operating expenses to produce high‐energy proton beams. In Japan, the cost of PBT for esophageal cancer is three times greater than that of IMRT.^29^ Additionally, there is a global‐regional imbalance in PBT clinical trials. East Asia, particularly China, has the highest incidence of esophageal cancer. However, the number of clinical trials investigating PBT for esophageal cancer is higher in Japan and the United States than in China. Consequently, the applicability of the trial results to all patients with esophageal cancer may be limited.

## 5 PROSPECTS OF PBT FOR THE TREATMENT OF ESOPHAGEAL CANCER

Flash radiation therapy, an ultra‐high‐dose‐rate radiation therapy, has the potential to revolutionize cancer treatment because it uses dose rates several orders of magnitude higher than that used in conventional clinical radiation therapy. Flash radiation therapy induces a flash effect using ultra‐high dose‐rate radiation to reduce normal tissue toxicity while maintaining local tumor control. However, most studies on flash radiation therapy have used electron beams with a low tissue penetration capacity, which limits their translation into clinical practice. Flash radiation therapy utilizing proton beams, which possess superior penetration capabilities, enables deeper tissue dose deposition, and maximizes therapeutic advantages.^30^ The key to the dosimetric advantages of PBT is the physical properties of the Bragg peak model. Shao et al.^31^ concluded that under the effect of a magnetic field in patients with tumors, the Bragg peak position of the proton beam can be adjusted to produce a uniform dose distribution to cover the tumor. Furthermore, they found that magnetic field‐modulated proton therapy (MMPT) effectively protected the surrounding vital organs. The MMPT maintains a low dose to the vital organs receiving PBT, with 95% of the dose line adequately covering the tumor. In pancreatic tumors, MMPT allows the proton beam to bypass the kidneys, delivering a uniform dose within the tumor. The esophagus is surrounded by mediastinal organs, and further development of MMPT holds promise for sparing the surrounding vital organs from irradiation without compromising the tumor dose coverage, thus maximizing the dosimetric benefits of PBT for esophageal cancer.

The development of laser‐accelerated proton beams is currently ongoing. Laser‐accelerated protons do not use synchrotrons or cyclotrons and therefore have low space and operating costs. However, laser‐accelerated protons exhibit a relatively low energy and reproducibility. When these issues are resolved, laser‐accelerated PBT may become widely used as linear accelerator facilities have replaced cobalt 60. If the cost of PBT is reduced, many patients can be treated, and clinical trials can be conducted.^32^ Additionally, magnetic resonance imaging (MRI) guidance is expected to enhance the targeting accuracy of PBT for mobile tumors. Integrating the soft tissue contrast and real‐time imaging capabilities of MRI with the precisely shaped dose distribution and optimal dose guidance offered by PBT is crucial to improving the precision of radiation therapy for esophageal cancer. However, to date, a hybrid system combining MRI and PBT has not been realized, owing to many technical challenges.^33^

## 6 HEAVY ION RADIATION THERAPY FOR ESOPHAGEAL CANCER

Heavy ions (particles heavier than electrons) particularly carbon ions are commonly used in radiation therapy. Carbon ions demonstrate a more significant dose decrease than protons in the longitudinal direction. Furthermore, owing to the increased mass of heavy ions, their greater inertia results in a smaller lateral scattering range, thereby maintaining directional stability when targeting tumors. The dose range is controlled with several times the precision of protons, allowing the Bragg peak to fall more accurately within the target volume. Additionally, nuclear interactions induced by heavy ions activate the irradiated tissue nuclei, resulting in focal radioactivity that can be imaged ex vivo. This radioactivity serves as an in vivo validation of dose deposition in both tumor and normal tissue. Heavy ions are charged particles that are deflected by magnetic fields. If a narrow beam of charged particles is deflected in the transverse direction, the radiation dose can “map” the entire contour of the tumor. During treatment, the energy and direction of the incident ions are adjusted to form an expanding Bragg peak, which accurately targets the tumor area, enabling three‐dimensional scanning of the beam to achieve precise shape and IMRT and minimize damage to the surrounding healthy tissues.^34^

The National Institute of Radiological Sciences (NIRS) in Chiba, Japan has extensive experience with heavy‐ion therapy, particularly carbon‐ion radiation therapy (CIRT). Since 1994, nearly 10,000 patients with cancer have been treated with CIRT. Clinical experience at NIRS has demonstrated the application of CIRT for the treatment of various solid tumors, including esophageal cancer.^35^, ^36^ Luo et al.^37^ demonstrated that carbon‐ion beams markedly decreased the viability of esophageal cancer cells and induced apoptosis in a dose‐dependent manner. Additionally, carbon‐ion beams induced G2/M phase cell cycle arrest in esophageal cancer cells, with pronounced G2 phase arrest observed at high doses, thereby inhibiting tumor metastasis in a dose‐dependent manner. The physical advantages of heavy‐ion beams, along with their unique biological effects on esophageal cancer cells, support our continued exploration of heavy‐ion therapy for esophageal cancer.

Although research on CIRT for esophageal cancer is still in its initial stages, the advantages of CIRT are significant, as shown in previous studies. In a retrospective study conducted by Isozaki et al.,^38^ CIRT was deemed a safe and effective approach for treating isolated lymph node recurrence following radical esophageal cancer treatment, with a reported 2‐year LRC rate of 92.4% and a 2‐year OS rate of 70.0%. Takakusagi et al.^39^ compared the radiation dose distribution of CIRT with those of 3D‐CRT and VMAT in stage I esophageal cancer treatment. The results showed that CIRT resulted in significantly lower radiation doses to the heart [D_mean_: (9.6±4.5) Gy vs (29.1±11.7) Gy vs (22.3±9.0) Gy], lungs [D_mean_: (1.8±0.9) Gy vs (5.3±1.8) Gy vs (11.4±2.3) Gy], spinal cord [D_max_: (25.6±3.5) Gy vs (44.6±0.6) Gy vs (41.3±2.9) Gy] and skin [D_max_: (15.1±9.5) Gy vs (30.1±4.6) Gy vs (24.2±10.3) Gy] compared to the other two modalities. These findings indicate a more favorable dose distribution of CIRT for esophageal cancer.

Similar to PBT, in CIRT for esophageal cancer, organ and target motions present a significant challenge in treatment planning. Treating moving targets with heavy‐ion irradiation, as well as irradiating stationary targets through moving tissues, remains technically challenging. Haefner et al.^40^ observed that target volume coverage was adequate for all configurations in the baseline CIRT program. However, slight cardiac deformation on 4D‐CT results in a significant dose overshoot, which increases the maximum dose (D_max_) in the spinal cord from the expected prescription dose of 16.7% to 60%. In the future, comprehensive treatment planning and exercise management techniques need to be developed and validated through multiple clinical trials to improve the effectiveness of heavy‐ion therapy while minimizing its toxic effects.

New therapeutic strategies are needed to further improve therapeutic efficacy. Kano et al.^41^ discovered that the histone deacetylase inhibitor cyclic hydroxamic‐acid‐containing peptide 31 (CHAP31) sensitized human esophageal squamous cell carcinoma to CIRT. in vivo experiments demonstrated that CHAP31 suppresses the expression of DNA repair‐related genes and significantly inhibits tumor growth in combination with CIRT.

Carbon ions can elicit immune responses in cancer cells; however, the underlying mechanisms are not fully understood. Du et al.^42^ compared the immune responses and underlying mechanisms in esophageal cancer cells following irradiation with X‐rays, protons, and carbon ions. The esophageal cancer cell line KYSE450 was irradiated with X‐rays, proton beams, and carbon ion beams at a dose of 15 Gy each. Subsequently, the cells were collected for RNA sequencing and gene enrichment analysis. Sequencing analysis revealed increased expressions of 2′‐5′‐oligoadenylate synthetase 1 (OAS1) a gene regulated by the interferon‐stimulated response element (ISRE) promoter and human leukocyte antigen B‐site (HLA‐B) an MHC class I gene after 3 days. These genes are linked to biological pathways associated with innate immune responses. X‐ray, proton, and carbon ion irradiation elicited similar immune responses regulated by the STING‐STAT1 axis. This demonstrates that X‐rays and CIRT elicit part of the same basis for the immune response in patients and that combining carbon ions with immunotherapy may exert a more potent antitumor effect.

Future research should focus on compiling additional clinical data from phase II trials of CIRT for esophageal cancer, investigating post‐treatment biological effects, and developing stable dose calculations and control systems. These efforts are essential for establishing an optimal heavy‐ion therapy system for esophageal cancer and fully harnessing the unique advantages of this treatment modality.

## 7 CONCLUSION AND PROSPECTS

In summary, compared with photon‐based radiation therapy, both proton and heavy ion therapies offer unique dosimetric advantages for esophageal cancer, particularly in delivering high‐dose radiation precisely to the tumor site while sparing the surrounding normal organs and tissues. PBT for esophageal cancer has reached a stage of significant advancement, demonstrating substantial clinical efficacy and the potential for further technical refinements. In contrast, heavy‐ion therapy for esophageal cancer is still in its early developmental stages owing to cost and safety concerns. Future advancements in this technology are expected to be driven by extensive clinical trial data and the integration of artificial intelligence.

## CONFLICT OF INTEREST STATEMENT

The authors declare no conflict of interest.

## ETHICS STATEMENT

Not applicable.
